# A Rare Type of Myeloma: A Case of Monoclonal IgD-Lambda

**DOI:** 10.7759/cureus.81089

**Published:** 2025-03-24

**Authors:** Recep Kaya, Alperen Burak Kocabıyık, Erdal Kurtoğlu, Volkan Karakuş

**Affiliations:** 1 Internal Medicine, Antalya Training and Research Hospital, Antalya, TUR; 2 Hematology, Antalya Training and Research Hospital, Antalya, TUR

**Keywords:** immunoglobulin d multiple myeloma, light chain multiple myeloma, myeloma, serum immunofixation, serum protein electrophoresis (spep)

## Abstract

An immunoglobulin D-multiple myeloma (IgD-MM) case represents a rare subtype of MM that is often observed in male patients under 65 years of age with an advanced stage at the time of diagnosis. It exhibits an aggressive course and is associated with a short survival period. In IgD-MM, an increase in lambda light chain proteins is expected. Given that IgD and IgE heavy chains are not routinely assessed in standard serum and serum immunofixation electrophoresis (IFE) analyses in MM patients, such cases may be misclassified and monitored as light chain myeloma. Therefore, distinguishing IgD or IgE myeloma from light chain myeloma represents a critical differential diagnosis. This case highlights the necessity of including IgD and IgE in diagnostic evaluations to prevent misclassification as light chain myeloma.

## Introduction

In patients with multiple myeloma (MM), an increase in the production of monoclonal immunoglobulins (Ig) and free light chains is observed due to malignant plasma cells with abnormal proliferative characteristics, leading to the accumulation of M-protein types. Among M-protein types, IgG-MM is the most common, observed in 52% of cases, followed by IgA-MM at 21%, light chain type at 16%, and the rare IgD-MM type, which is seen in 1-2% of cases [1]. IgD-MM is more closely associated with acute kidney injury, hypercalcemia, and amyloidosis as distinguishing features. In laboratory findings, while the M-protein peak may appear indeterminate in protein electrophoresis, a predominance of lambda light chains is often observed in serum and urine immunofixation electrophoresis (IFE) [2]. In such cases, IgD-MM should always be considered. However, since IgD monoclonal antibody is not assessed in standard IFE testing, IgD clonality - a monoclonal gammopathy rarely seen in MM - may be misinterpreted as light chain myeloma due to its diagnostic challenges. In this report, we present a case of IgD-lambda MM, which is rare and differentiates itself from light chain myeloma.

## Case presentation

A 74-year-old male had been experiencing complaints of weakness, fatigue, drowsiness, and muscle weakness for the past month. His medical history was significant for hypertension, but he had no other comorbidities. There was no history of smoking or alcohol use, and no family history of hematologic diseases. Laboratory results revealed anemia, thrombocytopenia, acute kidney failure, hypercalcemia, and elevated sedimentation rate. The laboratory results are presented in Table 1.

**Table 1:** Laboratory results and reference ranges

Parameter	Result	Reference Range
Blood Urea Nitrogen (BUN)	36 mg/dl	8-20 mg/dl
Creatinine	1.74 mg/dl	0.81-1.44 mg/dl
Potassium	5.2 mmol/L	3.5-5.1 mmol/L
Calcium	10.8 mg/dl	8.8-10.6 mg/dl
Albumin	4 g/dl	3.5-5.2 g/dl
Lactate Dehydrogenase (LDH)	357 U/L	<248 U/L
Uric Acid	11.7 mg/dl	3.5-7.2 mg/dl
Leukocytes	4000/mm³	4000-11000/mm³
Hemoglobin	6.8 g/dl	12-16 g/dl (female), 13-18 g/dl (male)
Platelets	99 x 10³/µL	150-400 x 10³/µL
Erythrocyte Sedimentation Rate (ESR)	>140 mmol/hour	0-20 mmol/hour
IgG	277 mg/dl	700-1600 mg/dl
IgA	46 mg/dl	70-400 mg/dl
IgM	18 mg/dl	40-230 mg/dl
Kappa/Lambda Ratio	0.054	0.26-1.65
Kappa Light Chain	1.64 mg/dl	6.7-22.4 mg/dl
Lambda Light Chain	30.4 mg/dl	8.3-27 mg/dl

A monoclonal lambda band was observed in the serum and urine immunofixation electrophoresis (IFE) (Table 2).

**Table 2:** Serum IFE and urine IFE results IFE: immunofixation electrophoresis

Protein (Band)	Serum IFE	Urine IFE
T	2 distinct bands	Faint signal
G (IgG)	No distinct signal	No distinct signal
A (IgA)	No distinct signal	No distinct signal
M (IgM)	No distinct signal	No distinct signal
κ (Kappa)	No distinct signal	No distinct signal
λ (Lambda)	Very faint signal (weak band)	Strong signal (clear band)

**Figure 1:**
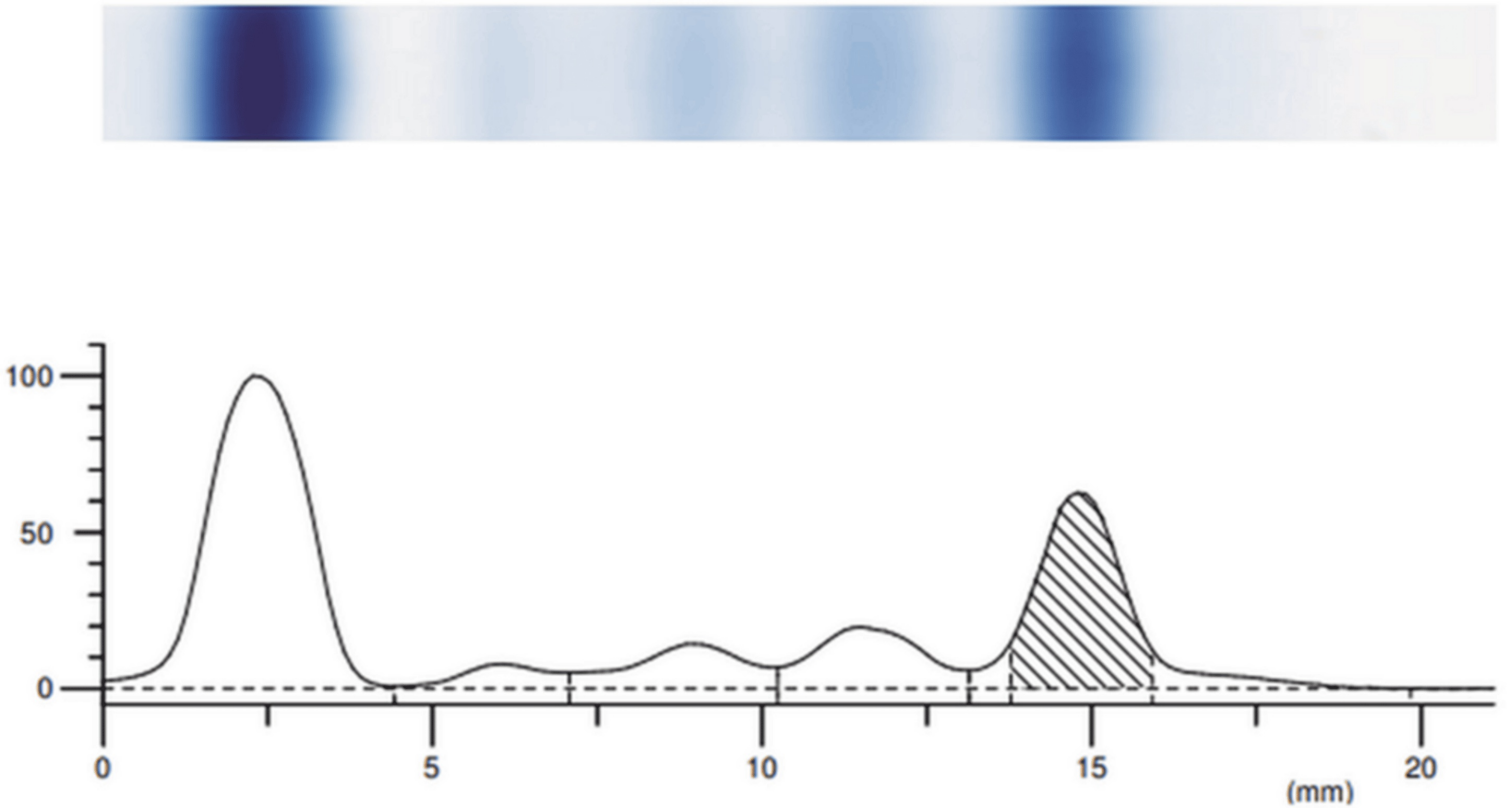
Protein electrophoresis The presence of an M-protein spike in the gamma band

Protein electrophoresis revealed a concentration of 1.85 (g/dl) of M-protein in the gamma band (Figure 1 and Table 3).

**Table 3:** Protein electrophoresis

Index	Band	Rel. Area (%)	Conc. (g/dL)	Range (g/dL)
1	Albumin	48.73 (L)	3.66	55.80 - 65.00
2	Alpha 1	3.44	0.26	2.20 - 4.60
3	Alpha 2	8.07 (L)	0.61	8.20 - 12.50
4	Beta	10.59	0.80	7.20 - 14.20
5	Gamma	29.17	2.19	11.50 - 18.60
Total			7.52	
5 M	Gamma M-spike 1	24.59%	1.85	
Ratio			0.49	

Due to the monoclonal lambda band observed in the serum and urine IFE and the M-protein peak in the gamma band, IgD and IgE were also tested in the serum IFE. The patient's IgD level was 25,500 IU/ml (<100 IU/ml), and IgD and lambda bands were observed in the serum IFE. Bone marrow biopsy pathology revealed that plasma cells accounted for 70% of the nucleated cells, and a diagnosis of monoclonal IgD-lambda multiple myeloma (MM) was established. Given the presence of acute kidney failure, the patient was initiated on a combination of bortezomib, lenalidomide, and dexamethasone was proposed.

## Discussion

IgD-MM is one of the rarest subtypes of multiple myeloma, accounting for approximately 2% of cases. It is observed in younger patients and at more advanced stages compared to other subtypes, and its survival rate is shorter [3]. IgD levels in serum are lower compared to other immunoglobulins (0-0.1 g/L), and due to the short half-life of IgD (2.8 days), the M-protein peak in electrophoresis is often minimal or undetectable [4]. In a study conducted by Selene et al. with 166 patients diagnosed with IgD-MM, 136 (81.9%) had the lambda light chain subtype, 30 (18.1%) had the kappa light chain subtype, and the median age of the patients was 54.5-65 years [5]. This study supports our findings by demonstrating a high prevalence of lambda light chain involvement in IgD-MM. While IgD-MM typically presents at an advanced stage in younger patients, with a poorer prognosis compared to other myeloma subtypes, in this case, the patient presented at an older age (74) with prodromal symptoms. In patients admitted for further investigation with suspected multiple myeloma, before diagnosing light chain MM, it is crucial to conduct advanced tests, including IgD and IgE testing in both serum and serum immunofixation electrophoresis. This is particularly important in cases where bands are detected exclusively in the light chain subtype, and a minimal or gamma M protein peak is observed in protein electrophoresis.

## Conclusions

In patients admitted with a preliminary diagnosis of multiple myeloma (MM), detailed laboratory results should exclude IgG and IgA MM, and in cases where light chain MM is suspected, IgD and IgE should always be screened in both serum and serum immunofixation electrophoresis. As mentioned earlier, due to the lower levels of IgD and IgE in serum, they may not be detectable in protein electrophoresis, leading to a misdiagnosis of light chain MM. This case underscores the necessity of incorporating IgD and IgE testing into standard diagnostic workups for suspected light chain myeloma to prevent misclassification. Therefore, in order to better understand the diagnosis and treatment process of rare forms of multiple myeloma, larger cohort studies are warranted to refine diagnostic and therapeutic strategies for IgD-MM.
